# The Role of Visualization in Estimating Cardiovascular Disease Risk: Scoping Review

**DOI:** 10.2196/60128

**Published:** 2024-10-14

**Authors:** Adrijana Svenšek, Mateja Lorber, Lucija Gosak, Katrien Verbert, Zalika Klemenc-Ketis, Gregor Stiglic

**Affiliations:** 1 Faculty of Health Sciences University of Maribor Maribor Slovenia; 2 Department of Computer Science Katholieke Universiteit Leuven Leuven Belgium; 3 Primary Healthcare Research and Development Institute Community Health Centre Ljubljana Ljubljana Slovenia; 4 Department of Family Medicine Faculty of Medicine University of Maribor Maribor Slovenia; 5 Department of Family Medicine Faculty of Medicine University of Ljubljana Ljubljana Slovenia; 6 Faculty of Electrical Engineering and Computer Science University of Maribor Maribor Slovenia; 7 Usher Institute University of Edinburgh Edinburgh United Kingdom

**Keywords:** cardiovascular disease prevention, risk factors, visual analytics, visualization, mobile phone, PRISMA

## Abstract

**Background:**

Supporting and understanding the health of patients with chronic diseases and cardiovascular disease (CVD) risk is often a major challenge. Health data are often used in providing feedback to patients, and visualization plays an important role in facilitating the interpretation and understanding of data and, thus, influencing patients’ behavior. Visual analytics enable efficient analysis and understanding of large datasets in real time. Digital health technologies can promote healthy lifestyle choices and assist in estimating CVD risk.

**Objective:**

This review aims to present the most-used visualization techniques to estimate CVD risk.

**Methods:**

In this scoping review, we followed the Joanna Briggs Institute PRISMA-ScR (Preferred Reporting Items for Systematic Reviews and Meta-Analyses extension for Scoping Reviews) guidelines. The search strategy involved searching databases, including PubMed, CINAHL Ultimate, MEDLINE, and Web of Science, and gray literature from Google Scholar. This review included English-language articles on digital health, mobile health, mobile apps, images, charts, and decision support systems for estimating CVD risk, as well as empirical studies, excluding irrelevant studies and commentaries, editorials, and systematic reviews.

**Results:**

We found 774 articles and screened them against the inclusion and exclusion criteria. The final scoping review included 17 studies that used different methodologies, including descriptive, quantitative, and population-based studies. Some prognostic models, such as the Framingham Risk Profile, World Health Organization and International Society of Hypertension risk prediction charts, Cardiovascular Risk Score, and a simplified Persian atherosclerotic CVD risk stratification, were simpler and did not require laboratory tests, whereas others, including the Joint British Societies recommendations on the prevention of CVD, Systematic Coronary Risk Evaluation, and Framingham-Registre Gironí del COR, were more complex and required laboratory testing–related results. The most frequently used prognostic risk factors were age, sex, and blood pressure (16/17, 94% of the studies); smoking status (14/17, 82%); diabetes status (11/17, 65%); family history (10/17, 59%); high-density lipoprotein and total cholesterol (9/17, 53%); and triglycerides and low-density lipoprotein cholesterol (6/17, 35%). The most frequently used visualization techniques in the studies were visual cues (10/17, 59%), followed by bar charts (5/17, 29%) and graphs (4/17, 24%).

**Conclusions:**

On the basis of the scoping review, we found that visualization is very rarely included in the prognostic models themselves even though technology-based interventions improve health care worker performance, knowledge, motivation, and compliance by integrating machine learning and visual analytics into applications to identify and respond to estimation of CVD risk. Visualization aids in understanding risk factors and disease outcomes, improving bioinformatics and biomedicine. However, evidence on mobile health’s effectiveness in improving CVD outcomes is limited.

## Introduction

### Background

Supporting and understanding the health of patients with chronic diseases remains a major challenge. Visualization has the potential to provide personalized and person-centered care [1]. The health data generated are often provided as feedback to patients, and visualization plays an important role in facilitating the interpretation and understanding of the data and, therefore, influencing their actions [2]. Visualization is being used to show patient outcomes in an increasing number of studies [3]. In addition, the review by Ooge et al [4] points to a lack of web-based visualization monitoring systems and systems aimed at laypeople. Visualization, such as pictures, sketches, charts, graphs, and diagrams, can help communicate health information usefully. Visualization can simplify the presentation of complex information and make it more appealing [5]. Algorithmic outcomes can typically be visualized in different ways, depending on the algorithm and the insights being sought. These insights are usually connected to health care activities, which more often focus on interpreting data rather than predicting or monitoring them [4]. These insights can be used to support both written and spoken health messages [5].

Digital health tools can help people with estimation of cardiovascular disease (CVD) risk by empowering or encouraging them to adopt healthier lifestyle habits using different techniques such as visualization to support actionable insights. This is a key public health strategy to prevent or treat CVDs [6]. In a study in which people were randomly selected, the Framingham Risk Profile (GFRP) decreased after 1 year for participants who saw visual imaging results and increased for the group that only saw the risk scores [7]. Some risk communication strategies such as percentages, bar graphs, and icon arrays, which provide patients with a probability, fail to increase risk perception [8,9]. Many of the most frequently used CVD risk scores, such as the GFRP, consider a 20% “risk of developing CVDs in the next 10 years” to be high. Because 20% appears in the lower part of the graph, these scores can be interpreted as low risk. The same is true for icon arrays, where many positive icons make it easy for patients to believe that they are unaffected [10,11]. CVD health assessment feedback is a method of presenting personalized risk information [12]. Providing additional evidence on CVD risk to individuals, such as that shown on heart scans or with a heart age above the individuals’ actual age, may provide a cue to action [13-15]. This is consistent with previous research where strategies using imaging or visualization were most useful in communicating personalized risk [16-18].

Several studies have demonstrated the potential of visualization tools not only in the estimation of CVD risk but also in influencing patient behavior. Turchioe et al [2] found that a line chart was the most used, particularly for data collected over a longer period. They found that patients had a better understanding of line graphs and bar graphs and that color effectively conveys risk, enhances comprehension, influences patient behavior, and boosts confidence in interpretation. Backonja et al [1] found that the use of colors and reference lines was helpful in interpreting data, which subsequently motivated patients to make healthier lifestyle choices. They also revealed that visualization provides many opportunities for explainable artificial intelligence in health care by providing insights into advanced algorithms through visualization, interaction, guidance, and direct explanations [4]. These findings underscore the importance of effective visualization in not only informing patients about their health status but also motivating them to take actionable steps to reduce their risk.

### Objectives

This review explored the potential benefits of visual interpretation for patients with CVDs, the world’s leading cause of death. Specifically, it aimed to explore how visualization techniques can influence patients’ understanding of their risk and motivate them to adopt healthier behaviors. This review focused on the impact of visual aids on risk perception and whether they lead to significant changes in lifestyle or treatment adherence in patients with CVDs.

## Methods

We followed the Joanna Briggs Institute PRISMA-ScR (Preferred Reporting Items for Systematic Reviews and Meta-Analyses extension for Scoping Reviews) guidelines to facilitate the analysis of different research methods [19] (Multimedia Appendix 1). The main objective of this review was to present the main visualizations for estimation of CVD risk and answer the following research question: “What types of visualizations (C) are used to estimate cardiovascular disease risk (P)?”

### Search and Search Strategy

The PubMed, CINAHL Ultimate (EBSCO), MEDLINE, and Web of Science databases were searched. The search also included gray literature from Google Scholar, where we did not review all the articles, only the highest-ranked ones, and included them according to the relevance of their content. We used the following search string: (“visualization” OR “visualisation tool*” OR “visual interpretation” OR “visual analytic*” OR “visualisation intervention*” OR “chart*” OR “data visualisation” OR “visualisation techniques” OR “visual representation”) AND (“cardiovascular disease* risk” OR “heart disease* risk” OR “cardiac disease* risk” OR “vascular disease* risk” OR “coronary heart disease* risk” OR “CVD risk”) (Textbox 1).

**Search strings for the databases.**

**PubMed**

- (“Visualization” OR “visualisation tool*” OR “visual interpretation” OR “visual analytic*” OR “visualisation intervention*” OR “chart*” OR “data visualisation” OR “visualisation techniques” OR “visual representation”) AND (“cardiovascular disease* risk” OR “heart disease* risk” OR “cardiac disease* risk” OR “vascular disease* risk” OR “coronary heart disease* risk” OR “CVD risk”) Filters: randomized controlled trial ([“Visualization” [All Fields] OR “visualisation tool*” [All Fields] OR “visual interpretation” [All Fields] OR “visual analytic*” [All Fields] OR “visualisation intervention*” [All Fields] OR “chart*” [All Fields] OR “data visualisation” [All Fields] OR “visualisation techniques” [All Fields] OR “visual representation” [All Fields]] AND [“cardiovascular disease risk” [All Fields] OR “heart disease risk” [All Fields] OR “cardiac disease risk” [All Fields] OR “vascular disease risk” [All Fields] OR “coronary heart disease risk” [All Fields] OR “CVD risk” [All Fields]]) AND (randomized controlled trial [Filter])

**CINAHL Ultimate**

- (“Visualization” OR “visualisation tool*” OR “visual interpretation” OR “visual analytic*” OR “visualisation intervention*” OR “chart*” OR “data visualisation” OR “visualisation techniques” OR “visual representation”) AND (“cardiovascular disease* risk” OR “heart disease* risk” OR “cardiac disease* risk” OR “vascular disease* risk” OR “coronary heart disease* risk” OR “CVD risk”)

**MEDLINE**

- (“Visualization” OR “visualisation tool*” OR “visual interpretation” OR “visual analytic*” OR “visualisation intervention*” OR “chart*” OR “data visualisation” OR “visualisation techniques” OR “visual representation”) AND (“cardiovascular disease* risk” OR “heart disease* risk” OR “cardiac disease* risk” OR “vascular disease* risk” OR “coronary heart disease* risk” OR “CVD risk”)

**Web of Science**

- (“Visualization” OR “visualisation tool*” OR “visual interpretation” OR “visual analytic*” OR “visualisation intervention*” OR “chart*” OR “data visualisation” OR “visualisation techniques” OR “visual representation”) AND (“cardiovascular disease* risk” OR “heart disease* risk” OR “cardiac disease* risk” OR “vascular disease* risk” OR “coronary heart disease* risk” OR “CVD risk”) (All Fields)

**Google Scholar**

- (“Visualization” OR “visualisation tool*” OR “visual interpretation” OR “visual analytic*” OR “visualisation intervention*” OR “chart*” OR “data visualisation” OR “visualisation techniques” OR “visual representation”) AND (“cardiovascular disease* risk” OR “heart disease* risk” OR “cardiac disease* risk” OR “vascular disease* risk” OR “coronary heart disease* risk” OR “CVD risk”)

### Eligibility Criteria

The review included articles published in English, the population included patients and research focusing on estimation of CVD risk, and the comparisons included different types of visualizations (related to digital health, mobile health, apps, images, charts, decision support systems, and other types of visualizations) for estimation of CVD risk. Only empirical studies were included.

Studies that did not involve patients or content about estimation of CVD risk or comparisons related to visualizations were excluded. Studies such as commentaries, editorials, and systematic and scoping reviews were excluded. We also excluded articles that were irrelevant and did not focus on the area under review (Textbox 2).

**Inclusion and exclusion criteria for the selected studies.**

**Inclusion criteria**

- Article type: empirical studies
- Language: English
- Comparison: visualizations (digital health, mobile health, mobile apps, images, charts, decision support systems, and other visualizations for estimating cardiovascular disease risk)
- Relevance: articles focused on the area under review

**Exclusion criteria**

- Article type: commentaries, editorials, and systematic and scoping reviews
- Language: other languages
- Comparison: research not including visualization comparisons
- Relevance: irrelevant articles not focused on the area under review

### Data Extraction

The search string retrieved in 14 results in PubMed, 495 in CINAHL Ultimate and MEDLINE, 265 in Web of Science and 2 in Google Scholar. In total, 2 authors analyzed the articles using the computer program Rayyan (Rayyan Systems Inc) [20]. Duplicate articles were removed before assessing their eligibility based on their titles and abstracts. If there was disagreement between the authors, a third author was consulted. The articles that passed this evaluation stage went through full-text analysis. We used the PRISMA (Preferred Reporting Items for Systematic Reviews and Meta-Analyses) flowchart [21] to describe the review process. In addition, 2 authors individually used the extraction algorithms using the standardized Joanna Briggs Institute data extraction tool [19] (Multimedia Appendix 1).

## Results

### Identified Studies

**Figure 1:**
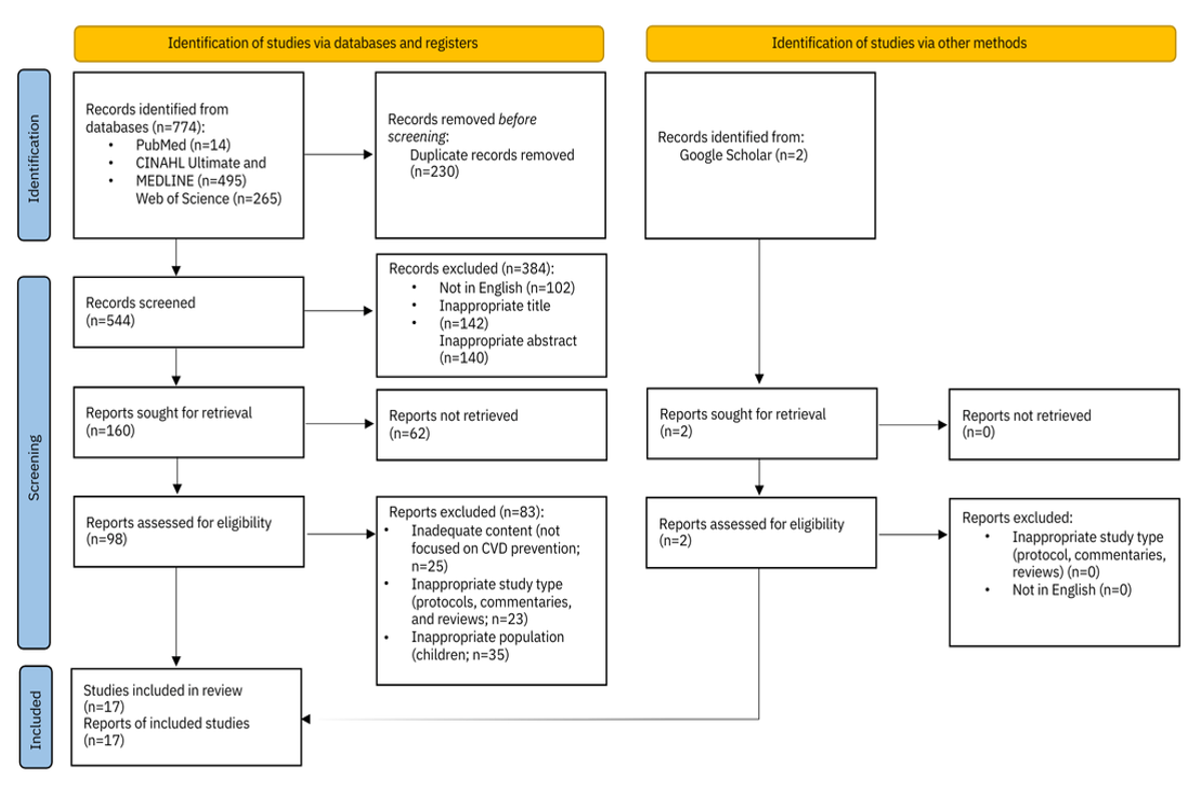
PRISMA (Preferred Reporting Items for Systematic Reviews and Meta-Analyses) flow diagram. CVD: cardiovascular disease.

First, we identified 774 records in the databases. After removing duplicates (230/774, 29.7%), we excluded records that were not in English (102/544, 18.8%) and had inappropriate titles (142/544, 26.1%) and abstracts (140/544, 25.7%). Then we get the reports (160/544, 29.4%) and records that could not be retrieved (62/160, 38.8%). In the next step, we excluded reports with inappropriate content (not focused on CVD prevention; 25/96, 26%), inappropriate study types (protocols; 23/96, 24%), and inappropriate study populations (children; 35/96, 36%). In addition, we reviewed only the highest-ranked results on Google Scholar, and we obtained 2 hits, which we included in the final analysis. A total of 17 studies were included in a scoping review (Figure 1 [22]).

Of the 17 identified studies, the most were from India (n=4, 24%), followed by the United States (n=4, 24%), Iran, Italy, and the United Kingdom (n=2, 12% each). Single studies were also identified by the authors from Oman, Australia, and Spain (each: 1/17, 6%). Descriptive study—model development (4/17, 24%) was the most used methodology, whereas quantitative studies and population-based longitudinal studies (1/17, 6%) were the least used methodology. The largest number of participants was found in the study by Bonner et al [13], which included 361,044 participants who used a heart age calculator. The study developed and validated a web-based heart age calculator. The smallest number of participants (N=70) was identified in the study by Fadel et al [23], which was an experimental study using visual analytics with a dashboard. All visualization methods were based on prognostic models for estimation of CVD risk.

The most used prognostic risk factors were age, sex, and blood pressure (16/17, 94%); smoking status (14/17, 82%); diabetes status (11/17, 65%); family history (10/17, 59%); high-density lipoprotein and total cholesterol (9/17, 53%); and triglycerides and low-density lipoprotein cholesterol (6/17, 35%). Other variables were used less frequently as predictors of CVD risk (Table 1).

We compared the results of the 17 studies on many different aspects. All the studies had the common aim of investigating the usefulness and comparability of the tools for estimation of CVD risk in different populations and settings. Most of the studies (12/17, 71%) were conducted among the general population, but some (5/17, 29%) focused on a target population of patients with different diseases (diabetes, rheumatoid arthritis, and hypertension, as well as patients using lipid-lowering therapy). However, they were different in terms of the specific purposes and contexts of their implementation. Some studies (4/17, 24%) focused on comparing ≥2 tools for the estimation of CVD risk [14,24,31,32], whereas others (13/17, 76%) examined the effect of a single tool for the estimation of CVD risk on the behavior, knowledge, decision-making, or quality of care of individuals or groups [13,23,29,37,38].

**Table 1:** Detailed information about the studies that included visualizations based on digital health.

Study	Methodology	Participants	Risk factors for CVD^a^	Prognostic model or clinical decision support system
Al-Lawati et al [24], Oman	Cohort study	1110 patients with DM2^b^	Age, gender, LDL-C^c^, total cholesterol, HDL-C^d^, triglyceride levels, age, FH^e^, blood pressure, smoking status, and diabetes status	Tools for estimation of CVD risk: the GFRP^f^ and the joint WHO^g^ and ISH^h^ risk prediction charts
Bonner et al [25], Australia	Descriptive study—model development	361,044 anonymous heart age calculator users (CVD risk factors only), 30,279 users who provided email addresses to request a report (heart age results), and 1303 survey respondents (psychological and behavioral questions)	Age, gender, FH of premature heart disease, smoking status, height, weight, diabetes status, blood pressure, cholesterol, and taking medication for high blood pressure	Web-based heart age calculator
Fadel et al [23], United States	Prospective quasi-experimental study	70 case simulations	Age, gender, LDL-C, total cholesterol, HDL-C, triglyceride levels, FH, blood pressure, smoking status, and diabetes status	Visual analytic dashboard—dashboard included graphical blood pressure trends with guideline-directed targets, calculated ASCVD^i^ risk score, and relevant medications; it also had recommendations and a treatment plan
Gidlow et al [14], United Kingdom	Qualitative study with quantitative process evaluation	240 participants (144 recorded consultations suitable for qualitative analysis and 48 video-stimulated recall interviews)	Age, gender, ethnicity, blood pressure, smoking status, diabetes status, HDL-C, and triglyceride levels	The JBS3^j^ lifetime risk calculator, with heart age, event-free survival age, and risk score manipulation
Gómez-Vaquero et al [26], Spain	Quantitative study	370 patients with a diagnosis of rheumatoid arthritis without history of CVD events	Age, gender, smoking status, total cholesterol and HDL, systolic and diastolic arterial blood pressure, and diabetes status	REGICOR^k^ app
Hassannejad et al [27], Iran	Population-based longitudinal study	6504 Iranian adults aged ≥35 years	Age, gender, systolic blood pressure, total cholesterol, diabetes status, FH, and WHR^l^	Web-based program and app (under preparation) based on the SPARS^m^ risk assessment chart
Kannan et al [28], India	Cross‑sectional study	217 participants between the ages of 32 and 90 years	Age, gender, LDL-C, total cholesterol, HDL-C, triglyceride levels, age, FH, blood pressure, smoking status, and diabetes status	WHO and ISH CVD risk prediction charts
Kavita et al [29], India	Quasi-experimental study	Validation of the intervention package: cardiology (n=2), community medicine (n=4), nursing (n=4), and fine arts (n=1); main study: 402 patients aged ≥40 years with hypertension were included	Age, gender, LDL-C, total cholesterol, HDL-C, triglyceride levels, age, FH, blood pressure, smoking status, and diabetes status	Risk communication package—it consisted of a booklet for nurses and a booklet and flash cards for patient education; nurses were trained to calculate 10-year absolute risk of CVD using the WHO and ISH risk prediction charts
Kowitt et al [30], United States	Cluster-randomized trial	The 28 practices included in the analyses represented 78,120 patients and 17,687 smokers	Blood pressure reduction medicine, statin prescription, aspirin use, and smoking status	Web education tools: HHN^n^—EHRs^o^ from clinical practices were used to create a practice-specific CVD population management dashboard (stratified sampling of patients aged 40 to 70 years using ASCVD risk scores)
Menotti et al [31], Italy	Descriptive study—model development	9 population studies in 8 Italian regions for a grand total of 17,153 participants (12,045 men and 5108 women) aged 35-74 years	Age, gender, systolic blood pressure, diabetes status, smoking status, BMI, HDL-C, LDL-C, and heart rate	Riskard 2005 chart and software
Menotti and Lanti [32], Italy	Descriptive study—model development	Data from Italian population study (Menotti et al [31]—17,153 participants)	Age, gender, systolic blood pressure, total serum cholesterol level, HDL-C level, and smoking status	Riskard HDL-C 2007 chart
Navar et al [33], United States	Cross-sectional study	7500 patients to be considered for lipid-lowering therapy from 175 cardiology, primary care, and endocrinology practices	Age, gender, LDL-C, total cholesterol, HDL-C, triglyceride levels, FH, 10-year CVD risk scores, and blood pressure	PALM^p^ registry mobile platform—custom-designed mobile platform that guides each participant from screening to informed consent to completion of surveys capturing patient-reported outcomes
Ordikhani et al [34], Iran	Cohort study	6504 participants aged 35 to 84 years	Age, gender, cholesterol, blood pressure, WHR, FH, diabetes status, and smoking status	XPARS^q^
Ordunez et al [35], United States	Descriptive study—model development	504 cases (84 cases for each of the 6 regions.	Age, gender, smoking status, systolic blood pressure, diabetes status, total cholesterol, and BMI	The HEARTS CVD risk calculator (CardioCal—iOS) app
Praveen et al [36], India	Cross-sectional study	Participants aged ≥40 years from 54 villages in South India; 62,194 individuals (84%) participated in the SMARThealth India study by Peiris et al [37]	Sociodemographic variables, age, gender, smoking status, diabetes status, total cholesterol, known chronic conditions and current drug treatments, and blood pressure; finger prick capillary blood glucose was estimated using a point-of-care device (Abbott FreeStyle Optium)	WHO and ISH charts
Peiris et al [37], India	Randomized controlled trial	Of the 11,484 people at high risk at baseline, 8642 (75.3%) were followed up on at the next 4 data collection points; an average of 120 per primary health center were included in the analysis	Age, gender, blood pressure, FH, smoking status, BMI, and glucose	Mobile health intervention—SMARThealth
Riley et al [38], United Kingdom	Mixed methods study	Participants aged ≥30 years who had completed the heart age test	Age, gender, ethnicity, postcode (to derive deprivation estimate), smoking status, weight, blood pressure, cholesterol level, FH, and other information about their current health status (eg, DM2 and rheumatoid arthritis)	Web-based health age tool based on the JBS3; the calculator’s algorithm uses QRISK^r^ data to estimate individual 10-year CVD risk, lifetime risk, and heart age

^a^CVD: cardiovascular disease.

^b^DM2: type 2 diabetes mellitus.

^c^LDL-C: low-density lipoprotein cholesterol.

^d^HDL-C: high-density lipoprotein cholesterol.

^e^FH: family history.

^f^GFRP: Framingham Risk Profile.

^g^WHO: World Health Organization.

^h^ISH: International Society of Hypertension.

^i^ASCVD: atherosclerotic cardiovascular disease.

^j^JBS3: the Joint British Societies recommendations on the prevention of cardiovascular disease.

^k^REGICOR: Framingham-Registre Gironí del COR**.**

^l^WHR: waist-to-hip ratio.

^m^SPARS: simplified Persian atherosclerotic cardiovascular disease risk stratification.

^n^HHN: Heart Health Now.

^o^EHR: electronic health record.

^p^PALM: Provider Assessment of Lipid Management.

^q^XPARS: Explainable Persian Atherosclerotic Cardiovascular Disease Risk Stratification.

^r^QRISK: Cardiovascular Risk Score.

### Prognostic Models

The studies used different tools and prognostic models to estimate CVD risk based on different factors and parameters. Some prognostic models were simpler and did not require laboratory tests, such as the GFRP [24], World Health Organization (WHO) and International Society of Hypertension (ISH) risk prediction charts [24,28,29,36], Cardiovascular Risk Score (QRISK2), and a simplified Persian atherosclerotic CVD risk stratification (SPARS) [27], whereas others were more complex and required laboratory tests, such as the Joint British Societies recommendations on the prevention of CVD (JBS3) [14], Systematic Coronary Risk Evaluation (SCORE), and Framingham-Registre Gironí del COR (REGICOR) [26]. Some tools and prognostic models were designed for estimation of CVD risk in the short term (eg, 10 years), such as the GFRP, WHO and ISH, QRISK2, SCORE, and REGICOR [14,24,26,39], whereas others were designed for estimation of CVD risk in the long term, such as the JBS3 and SPARS [27]. Some tools presented the estimation of CVD risk as a number (GFRP, WHO and ISH, QRISK2, SCORE, and REGICOR), whereas others as visual elements, such as cardiac age [14,25,27,38] and estimation of CVD risk [26].

Technology-based interventions have been shown to increase the usefulness of tools for the estimation of CVD risk and can affect several outcomes, such as increasing users’ knowledge, perception, motivation, intention, self-efficacy, satisfaction, compliance, and quality of care regarding their CVD risk and suggesting potential actions to reduce it; changing users’ behavior, lifestyle, risk factors, biological parameters, clinical outcomes, and overall CVD risk to obtain better outcomes; and improving clinical staff’s performance, job satisfaction, confidence, communication, decision-making, and quality of care when the estimation of CVD risk tools (Table 2).

Some of the studies we reviewed (12/17, 71%) used technology-based interventions to improve the effect of tools for the estimation of CVD risk on participants’ behavior, knowledge, decision-making, or quality of care. These interventions took the form of charts, tables, and diagrams (9/17, 53%) and apps (3/17, 18%). These interventions had different characteristics such as 1. presentation formats: displaying CVD risk in different formats, such as numbers, colors, and graphs; 2. user interactivity: allowing users to influence the estimation of their CVD risk by entering or modifying their own data, such as blood pressure, cholesterol, smoking status, physical activity, diet, and more. 3. Different types of tools and systems: clinical decision support systems (3/17, 18%), dashboards (2/17, 12%), education tools (3/17, 18%), and web-based tools (4/17, 24%) and software (2/17, 12%); 4. User engagement: providing feedback, advice, encouragement, reminders, goals, plans, support, or guidance to users based on the estimation of their CVD risk and needs; In some of the articles (6/17, 35%), the same authors described multiple different types of visualizations for estimating CVD risk, rather than focusing on just one type. This facilitating communication, collaboration, coordination, or shared decision-making between users and clinical staff or between users and other users. The most used format to display data in the studies was “visual cues” (10/17, 59%), followed by “bar charts” (5/17, 29%) and “graphs” (4/17, 24%; Table 3).

**Table 2:** Summary table for studies using visualization as a digital intervention.

Study	Duration of the intervention	Model of delivery	Outcome or outcomes
Al-Lawati et al [24]	January 2008 to December 2008	Several tools for estimation of CVD^a^ risk in the form of equations or charts were produced to assist clinicians in making intervention decisions for the primary prevention of CVDs	The GFRP^b^ tool found more patients than the joint WHO^c^ and ISH^d^ tool at 10-year CVD risk levels of 10% to <20% and 20% to <30%. At CVD risk levels of ≥30%, both tools found similar numbers of patients (22% vs 24%; *P*=.12). The GFRP tool also showed that almost twice as many men at CVD risk levels of ≥10% required aspirin treatment compared with the WHO and ISH charts (86% vs 43%).
Bonner et al [25]	Follow-up to support behavior change over a 10-week period	The user’s heart age was displayed as a result and compared to their actual age to see whether it was younger, the same, or older. This was repeated after 10 weeks.	The study showed the psychological and behavioral results of the people who responded to the survey. Most of them (892/1303, 68.46%) remembered their heart age category correctly 10 weeks after receiving their first result. They knew whether their heart age was younger than, the same as, or older than their actual age. People who had younger (104/155, 67.1%) or older (735/1055, 69.67%) heart age results also remembered their heart age correctly, but it was significantly lower for people who had the same heart age results (53/93, 57%).
Fadel et al [23]	Primary care clinicians to participate over a 2-month period	Use of the dashboard with the EHR^e^ compared with use of the EHR alone	Using visual analytics to extract important data from the EHR and presenting them in a clear and useful way can help physicians work better. Using a visual dashboard to display key data from the EHR and reduce chart review time can also help improve the quality of evidence-based care in primary care.
Gidlow et al [14]	Data collection took place from January 2017 to February 2019	Participants received a health check using either the usual QRISK2^f^ calculator, which estimates the 10-year risk of CVD, or the JBS3^g^ calculator, which shows the estimation of CVD risk with manipulation of heart age, event-free survival age, and risk score.	The health check took different amounts of time (from 6.8 to 38 minutes), but most of them were brief (60% took <20 minutes), with very little discussion about estimation of CVD risk (on average <2 minutes). The JBS3 calculator, which shows the estimation of CVD risk with heart age, event-free survival age, and risk score manipulation, led to more conversations about CVD risk and less practitioner-controlled consultations than the QRISK2 calculator, which estimates CVD risk.
Gómez-Vaquero et al [26]	—^h^	CVD risk index was calculated according to data on the age at the time of the study.	There was no clear difference between the SCORE^i^ and REGICOR^j^ indexes in how well they estimated the CVD risk for the Spanish population without rheumatic diseases according to the comparisons. The SCORE and REGICOR indexes estimate CVD mortality and CVD events, respectively, and none of them has been tested for the prediction of subclinical atherosclerosis.
Hassannejad et al [27]	Follow-up for at least 10 years	SPARS^k^ chart	Both the nonlaboratory and laboratory models agreed on the risk levels of patients and correctly classified them. The models also performed well in external validation, with similar Harrell C values of 0.77 (95% CI 0.75-0.78) for the nonlaboratory model and 0.78 (95% CI 0.76-0.79) for the laboratory model. This approach can provide a simple tool for risk assessment in cases in which laboratory testing is unavailable, inconvenient, or costly.
Kannan et al [28]	Period of 2 months from September 2018 to October 2018	Standard examination and questionnaire and quick education, including adherence to medication, diet, physical activity, addictions, and stress management, were administered to all the participants.	The study showed that, of 217 participants, 30 (14%) had moderate to high risk (>20%) of CVD. The CVD risk pattern showed that, of 216 people, 141 (65%) had a low risk (<10%), 46 (21.2%) had a mild risk (10%-20%), 21 (9.7%) had a moderate risk (20%-30%), and 9 (4.1%) had a high risk (>40%), with men being more susceptible than women. The WHO and ISH chart was identified as a simple and low-cost method for screening.
Kavita et al [29]	Follow-up at the 1st, 3rd, and 6th months telephonically to reinforce risk reduction and then on the 12th month using the WHO and ISH chart	The authors developed a specific risk communication package that included visual aids such as charts and tables to better present CVD risk estimates to study participants. Visualization was used as part of the intervention to improve understanding of risk and encourage participants to make healthy behavior changes.	The study found that the nurse-led intervention was effective in modifying risk and improving treatment usefulness among participants. In the primary prevention group, after 1 year of follow-up, there was a significantly higher number of participants in the low risk category (70%) than in the baseline estimate (60.6%). In the secondary prevention group, the mean treatment usefulness score for participants in the intervention group (7.60) was significantly higher than that for the comparison group (5.96), with a large effect size of 1.1. These results suggest that visualization can be a useful tool to improve understanding and communication of CVD risk and encourage patients to adopt healthy behaviors.
Kowitt et al [30]	The intervention began in January 2016 and ended in November 2017. Follow-up was before the intervention, 6 months after the intervention, and 12 months after the intervention start	Practices’ EHRs were used to create a practice-specific CVD population management dashboard; charts and educational tools such as web-based modules, live webinars, and occasional face-to-face collaborative meetings	An intervention targeting multiple risk factors for estimation of CVD risk in several small primary care clinics was useful in increasing the frequency of tobacco screening and smoking cessation support. A significant and meaningful number of smokers may have quit because of the intervention. It is unclear which component of the intervention improved tobacco screening and cessation support outcomes or whether the components of the intervention worked synergistically to improve these outcomes.
Menotti et al [31]	—	Riskard 2005 chart and software—for people with no history of similar clinical conditions, the Riskard 2005 table can be used to estimate the likelihood of having a first CVD event (as defined previously) in 10 years.	The different risk factors have multivariate coefficients that are as expected and have high values, and they discriminate well between outcomes. The software provides more accurate estimates than the chart, and considers factors that the chart does not.
Menotti and Lanti [32]	—	A chart accommodating sex, age, total cholesterol level, HDL-C^l^ level, systolic blood pressure, and cigarette consumption was subsequently produced.	A educational role of charts. The first chart for estimation of CVD risk in Italy that included HDL-C The models showed good predictive power, with approximately 30% of events in the top decile of estimated risk and approximately 50% in the top quintile of estimated risk.
Navar et al [33]	—	The PALM^m^ registry—the app evaluates how well patients estimate their own risk of CVD and how different ways of presenting CVD risk may lead to qualitative differences in patient-perceived risk and receptiveness to treatment.	PALM is an electronic platform that is easy to use and reduces the burden and errors of data entry. It also protects the data from being lost by regularly uploading them web-based. The survey is digital and can be tailored to participants’ responses; for example, adults who have stopped taking statins can be asked about their previous side effects, whereas adults who have never taken statins can be asked about their willingness to start. The electronic format also makes it easy to randomize participants to different risk communication scenarios in the patient survey. The visual aids used are Cates plots and bar graphs.
Ordikhani et al [34]	At the beginning of 2001 and then repeated in 2007 and 2011 using the same methods	Chart-based models for CVD risk and chromosome representation; 2D representation in 1 risk chart called XPARS^n^	A total of 5432 study participants who did not have CVDs at baseline; 705 developed CVDs within 10 years of follow-up. XPARS with only 4 features was considered, which is much easier to use as the chart is simpler and there is no need for laboratory cholesterol measurement. Proposed method using PARS^o^ model had the highest accuracy among those in the article, where it attained an AUROC^p^ of 0.74 with 8 features. Using the same features and, as a result, the same number of cells, XPARS could improve the AUROC to 0.76.
Ordunez et al [35]	—	The HEARTS CVD risk calculator	Providing high-quality care in primary care settings, which can help prevent CVDs, is a key goal of the HEARTS app. The HEARTS app is an important achievement in the effort to reduce the burden of avoidable CVDs in the Americas. This risk stratification scheme is aligned with the WHO’s recommendations for the management of CVD risk.
Praveen et al [36]	Between February 2014 and May 2014	WHO and ISH charts—evaluating an intervention aimed at improving CVD risk management	A total of 4 out of every 100 people in the study had already been diagnosed with CVDs. After estimating pretreatment blood pressure levels in patients already on medication, 11.8% had hypertension with a blood pressure cutoff at 160/100 mm Hg, and 29.9% had hypertension with a blood pressure cutoff at 140/90 mm Hg. A total of 12,230 individuals (19.6%) were taking blood pressure–lowering medication at the time of data collection.
Peiris et al [37]	Follow-up care over a 6-month period	SMARThealth intervention—gather important health information; tell the person their risk level; give advice on how to improve their lifestyle through exercise, diet, and avoiding tobacco and alcohol; and refer high-risk patients to the physician at the primary health center. The intervention consisted of (1) community health workers who visit households and assess CVD risk using a mobile device, (2) electronic referral and advice for primary health center physicians, and (3) a system to track follow-up care.	In the high-risk subgroup, there was more use of antihypertensive medication during the intervention period (54.3% vs 47.9%; OR^q^ 1.22, 95% CI 1.03-1.44) but no effect on blood pressure control. A total of 85.9% of the baseline group were screened, and 70% of all high-risk referrals were followed up on. There were no differences between the intervention and control groups in the proportion of people achieving target blood pressure (41.2% vs 39.2%; adjusted OR 1.01, 95% CI 0.76-1.35) or receiving antihypertensive medication.
Riley et al [38]	Data collection was conducted on the web from January 2021 to March 2021	Participants took the heart age test and then answered questions about how they felt and how the test affected them, what they planned to do next, and their demographic characteristics. A telephone interview was conducted to talk about their experience and the effect of the tool on future behavior intentions.	The results showed that, with approximately 5 million people having completed the health age test by June 2020, people were very interested in their heart health. Compared to percentage risk scores, there is evidence that heart age is more emotionally impactful and improves risk perception and recall. Nevertheless, most participants said that they would recommend the heart age tool to others, had recommended it to others, and would take the test again in the future and self-reported that they had made or intended to make changes in their health behavior (eg, lose weight, be more active, or eat in a healthier way) or had been encouraged and motivated by the test to maintain the changes in their health behavior.

^a^CVD: cardiovascular disease.

^b^GFRP: Framingham Risk Profile.

^c^WHO: World Health Organization.

^d^ISH: International Society of Hypertension.

^e^EHR: electronic health record.

^f^QRISK2: Cardiovascular Risk Score.

^g^JBS3: the Joint British Societies recommendations on the prevention of cardiovascular disease.

^h^Not applicable.

^i^SCORE: Systematic Coronary Risk Evaluation.

^j^REGICOR: Framingham-Registre Gironí del COR.

^k^SPARS: simplified Persian atherosclerotic cardiovascular disease risk stratification.

^l^HDL-C: high-density lipoprotein cholesterol.

^m^PALM: Provider Assessment of Lipid Management.

^n^XPARS: explainable Persian atherosclerotic cardiovascular disease risk stratification.

^o^PARS: Persian atherosclerotic cardiovascular disease risk stratification.

^p^AUROC: area under the receiver operating characteristic curve.

^q^OR: odds ratio.

**Table 3:** An overview of the technology and data display formats used in the studies (N=17).

	Visual cues^a^	Bar chart^b^	Graphs	Specific graphs^c^	Line graphs^d^	Cates plot^e^	Pie chart^f^	Flat chart^g^	Timeline^h^	Matrix^i^
Al-Lawati et al [24]	✓									
Bonner et al [25]				✓						
Fadel et al [23]		✓	✓		✓				✓	
Gidlow et al [14]								✓		
Gómez-Vaquero et al [26]	✓									
Hassannejad et al [27]		✓								
Kannan et al [28]	✓									
Kavita et al [29]	✓		✓							
Kowitt et al [30]				✓						
Menotti et al [31]	✓									
Menotti and Lanti [32]	✓									
Navar et al [33]		✓				✓				
Ordikhani et al [34]	✓									✓
Ordunez et al [35]	✓	✓								
Praveen et al [36]	✓									
Peiris et al [37]	✓	✓	✓		✓		✓			
Riley et al [38]			✓							
Total, n (%)	10 (59)	5 (29)	4 (24)	2 (12)	2 (12)	1 (6)	1 (6)	1 (6)	1 (6)	1 (6)

^a^Elements such as colors, symbols, or markers that help interpret the data.

^b^Graphs displaying data as vertical or horizontal bars, where the length or height of each bar represents the value.

^c^Types of graphs designed for particular purposes (eg, heat maps, Sankey diagrams, and network graphs).

^d^Graphs that plot data points connected by straight lines, often used to show trends over time.

^e^A specific type of plot used to visualize certain types of data, common in medical research.

^f^A circular chart divided into segments, each representing a proportion of the whole.

^g^A simple chart that presents data without additional dimensions or complexities.

^h^A graphical representation of events or data in chronological order.

^i^A grid layout displaying data in rows and columns, allowing for comparisons across different variables.

## Discussion

### Principal Findings and Comparison With Prior Work

We reviewed and compared the results of 17 studies that investigated the usefulness and comparability of different tools for the estimation of CVD risk. These studies were conducted in different countries and contexts, such as Oman, Australia, the United States, the United Kingdom, Spain, and Iran. The tools for the estimation of CVD risk used in these studies were in the form of equations, tables, graphs, or computer programs, such as GFRP, WHO and ISH, QRISK2, JBS3, SCORE, REGICOR, and SPARS. The results of these studies showed differences and similarities between tools for the estimation of CVD risk in terms of their objectives, methods, criteria, results, and limitations.

Of the studies reviewed, we found that only the dashboards by Fadel et al [23], Gidlow et al [14], and Hassannejad et al [27] included a tool that also allowed for goal setting, where the health care professionals and patients can agree on target goals and then calculate and visualize how the risk will change over time if these goals are met and maintained. The latter contributes to a better understanding of the impact of lifestyle or treatment adherence. In addition, Mendez et al [40] suggest that these interactions in the visualization tools themselves can help inform patients about the estimation of CVD risk and improve patient understanding of risk and the potential impact of risk-reducing interventions, which we believe can help patients make more informed and empowered decisions to achieve greater risk reduction.

We also found studies that included cardiac imaging as an additional indicator of CVD risk [41-43]. A randomized controlled trial by Whitmore et al [44] with a sample of 7000 patients showed that cardiac imaging not only helped with CVD diagnosis and estimation of CVD risk but also, importantly, helped educate and motivate people to engage in risk modification or lifestyle changes. This highlights the critical role of diagnostic tools not only in clinical decision-making but also in improving patient compliance with treatment and promoting sustainable lifestyle changes that are essential for long-term CVD health outcomes. An analysis of the visualization techniques used in the different studies showed that the most used visualization type was color plots. Colors are important because, together with warning words, they can attract more attention from users [45]. Color coding in matrices and graphs usually reflects the level of risk [46]. This can be particularly valuable in medical settings, where visual cues can improve patients’ understanding of their health risks, potentially leading to better adherence to treatment recommendations and lifestyle changes. In addition, effective visualizations can simplify complex data, making them more accessible to a wider audience, including patients with varying levels of health literacy. On the other hand, it is important to bear in mind that some patients may have color vision impairment, which may affect the interpretation of the data. It is also important to consider the diversity of cultural backgrounds as colors have different meanings in different environments [47].

Bar charts were the second most common type of visualization, followed by graphs. Less commonly used were special graphs and line graphs. On the other hand, Cates plots, pie charts, level charts, timelines, 3D models, infographics, and matrices were used only once each. This finding indicates the predominance of colored visuals in the presentation of data, which may help improve readers’ understanding and perception of the information. In contrast, a study by van Weert et al [48] found that most participants preferred to see risk in the form of hourly, pie, or bar charts. They also found that younger age, higher mathematical ability, and higher graphical literacy contribute to higher knowledge and understanding of risk scores. This suggests that, while certain visual formats may be more appealing or accessible to the general population, individual differences in cognitive abilities, such as numeracy and familiarity with graphical representations, play an important role in the effectiveness of these visual aids. Therefore, tailoring risk communication to the abilities of the user may enhance understanding and improve decision-making regarding health interventions and risk management.

### Limitations

We found that tools for the estimation of CVD risk can be useful for a variety of purposes and contexts, but they also have some limitations that need to be considered when using and interpreting them. One of the limitations of this paper is that we did not include machine learning classification approaches, which offer important advantages in predicting and classifying outcomes but whose limitations should be considered. Future research should aim to address these limitations by incorporating diverse datasets and using methods that increase the transparency and interpretability of models. We recommend that the selection of tools for the estimation of CVD risk should consider the characteristics of the target population, the availability and quality of the data, the way in which risk is presented, the interaction between users and the tools, and other factors that may affect the tools’ performance and comparability. We also suggest that tools for the estimation of CVD risk should be regularly updated, validated, and calibrated to ensure their accuracy, reliability, and generalizability. Ongoing advancements in machine learning techniques and data collection methods will contribute to more accurate and reliable risk predictions in the future. We hope that this paper will contribute to a better understanding and use of tools for the estimation of CVD risk in practice and research.

### Conclusions

We identified some innovative features of tools, such as goal setting, visualization, and cardiac imaging, that could improve the estimation of CVD risk and user engagement in risk reduction. We conclude that the selection of tools for the estimation of CVD risk should be based on several factors, such as the characteristics of the target population, the availability and quality of the data, the display and interaction with risk, and the performance and comparability of the tools. We also recommend that tools for the estimation of CVD risk should be regularly updated, validated, and calibrated to ensure their accuracy, reliability, and generality. Future research should test visualization tools to determine their potential impact on patients and their usefulness for health care professionals.

## Appendix

Multimedia Appendix 1 PRISMA (Preferred Reporting Items for Systematic Reviews and Meta-Analyses) checklist.

## Abbreviations

CVD: cardiovascular disease
GFRP: Framingham Risk Profile
ISH: International Society of Hypertension
JBS3: the Joint British Societies recommendations on the prevention of cardiovascular disease
PRISMA: Preferred Reporting Items for Systematic Reviews and Meta-Analyses
PRISMA-ScR: Preferred Reporting Items for Systematic Reviews and Meta-Analyses extension for Scoping Reviews
QRISK2: Cardiovascular Risk Score
REGICOR: Framingham-Registre Gironí del COR
SCORE: Systematic Coronary Risk Evaluation
SPARS: simplified Persian atherosclerotic cardiovascular disease risk stratification
WHO: World Health Organization
